# Supplementary material for: Global diversity of soil-transmitted helminths reveals population-biased genetic variation that impacts diagnostic targets
Source: Nat Commun. 2025 Jul 10;16:6374. doi: 10.1038/s41467-025-61687-0 (PMC12246136; doi:10.1038/s41467-025-61687-0)
Supplement: Supplementary file 3 — Description of Additional Supplementary Files [file 41467_2025_61687_MOESM3_ESM.pdf]

Inventory of Additional Supplementary Data Files

File name: Supplementary Data 1
Description: Country of origin, read counts and accession number for all faecal, egg, and worm samples included in the study

File name: Supplementary Data 2
Description: Whole genome and mitochondrial genome assemblies used as references in this study

File name: Supplementary Data 3
Description: Number of single-nucleotide polymorphisms (SNPs) and number of individuals (shown as SNPS/individuals) in mitochondrial genomes retained after each step of filtering

File name: Supplementary Data 4
Description: List of nuclear repeat- and ribosomal-based qPCR diagnostic targets

File name: Supplementary Data 5
Description: Genome coordinates of diagnostic targets per species

File name: Supplementary Data 6
Description: Primer and probe binding sites for repeat diagnostic targets across genomes of *Ascaris lumbricoides*, *Trichuris trichiura* and *Necator americanus*

File name: Supplementary Data 7
Description: Number of single-nucleotide polymorphisms (SNPs), repeats, and individuals retained after each filtering step (as SNPs/repeats/individuals)

File name: Supplementary Data 8
Description: List of wild-type (without SNP) and mutated (with SNP - in red) repeat targets tested by qPCR in this study

File name: Supplementary Data 9
Description: Sample codes, countries of origin, and extraction kits of samples sequenced as part of this study
