# Supplementary material for: Global diversity of soil-transmitted helminths reveals population-biased genetic variation that impacts diagnostic targets
Source: Nat Commun. 2025 Jul 10;16:6374. doi: 10.1038/s41467-025-61687-0 (PMC12246136; doi:10.1038/s41467-025-61687-0)
Supplement: Supplementary file 13 — Reporting Summary [file 41467_2025_61687_MOESM13_ESM.pdf]

Corresponding author(s): Marina Papaiakovou
Stephen R Doyle

Last updated by author(s): 13/06/2025

Reporting Summary

Nature Portfolio wishes to improve the reproducibility of the work that we publish. This form provides structure for consistency and transparency in reporting. For further information on Nature Portfolio policies, see our Editorial Policies and the Editorial Policy Checklist.

Statistics

For all statistical analyses, confirm that the following items are present in the figure legend, table legend, main text, or Methods section.

n/a	Confirmed	
☐	☒	The exact sample size (*n*) for each experimental group/condition, given as a discrete number and unit of measurement
☐	☒	A statement on whether measurements were taken from distinct samples or whether the same sample was measured repeatedly
☐	☒	The statistical test(s) used AND whether they are one- or two-sided<br>*Only common tests should be described solely by name; describe more complex techniques in the Methods section.*
☒	☐	A description of all covariates tested
☒	☐	A description of any assumptions or corrections, such as tests of normality and adjustment for multiple comparisons
☐	☒	A full description of the statistical parameters including central tendency (e.g. means) or other basic estimates (e.g. regression coefficient) AND variation (e.g. standard deviation) or associated estimates of uncertainty (e.g. confidence intervals)
☐	☒	For null hypothesis testing, the test statistic (e.g. *F*, *t*, *r*) with confidence intervals, effect sizes, degrees of freedom and *P* value noted<br>*Give P values as exact values whenever suitable.*
☒	☐	For Bayesian analysis, information on the choice of priors and Markov chain Monte Carlo settings
☒	☐	For hierarchical and complex designs, identification of the appropriate level for tests and full reporting of outcomes
☒	☐	Estimates of effect sizes (e.g. Cohen's *d*, Pearson's *r*), indicating how they were calculated

Our web collection on statistics for biologists contains articles on many of the points above.

Software and code

Policy information about availability of computer code

Data collection	Sequencing data (150 bp paired-end for n=150 samples) were generated using an Illumina NovaSeq 6000 (Novogene, Hong Kong).
Data analysis	Custom code to analyse data and reproduce the figures presented is open and freely available at https://github.com/MarinaSci/Global_skim_analysis/ and archived under a stable DOI at Zenodo (Papaiakovou, 2025): https://doi.org/10.5281/ZENODO.15639714.<br>Trimmomatic, v.0.39-2<br>BWA-MEM (v.0.7.17-r1188)<br>samtools view (v.1.6)<br>samclip (v.0.4.0)<br>sambamba (v.0.8.2)<br>Picard MarkDuplicates (v.picard-2.18.29-0)<br>bedtools multicov (v2.30.0)<br>UpSetR (v.1.4.0) package in R (v.4.2.2)<br>bcftools mpileup (v.1.16)<br>pcaMethods (v.pcaMethods_1.90.0)<br>Grenedalf (v.0.3.0)<br>pixy (v.1.2.7.beta1)<br>nucmer (mummer package, v.3.23)<br>ClustalOmega (msa package, v.1.30.1, R)<br>Heatmap.2 (package ComplexHeatmap, v.2.15.4) in R

bedtools coverage (v2.30.0)
bedtools makewindows (v2.30.0)
bedtools getfasta (v2.30.0)
seqkit locate (v.2.7.0)
bedtools intersect (v2.30.0)
BUSCO (v.5.6.1)
R (v.4.2.2)

For manuscripts utilizing custom algorithms or software that are central to the research but not yet described in published literature, software must be made available to editors and reviewers. We strongly encourage code deposition in a community repository (e.g. GitHub). See the Nature Portfolio guidelines for submitting code & software for further information.

Data

Policy information about availability of data

All manuscripts must include a data availability statement. This statement should provide the following information, where applicable:

- Accession codes, unique identifiers, or web links for publicly available datasets
- A description of any restrictions on data availability
- For clinical datasets or third party data, please ensure that the statement adheres to our policy

Study and sample accessions for the sequencing data analysed in this study (n=850 datasets) are described in Supplementary Data 1 and are available from ENA. The sequencing data that were sequenced de novo in this study (n=150) have been deposited in the European Nucleotide Archive (ENA) under project accession code PRJEB90452 [https://www.ebi.ac.uk/ena/browser/view/PRJEB90452] with a release date of the 13th June 2025. Reference genomes used for read mapping and comparative genomics are described in Supplementary Data 2. A list of all the publicly available datasets (from ENA) used in this study is provided in the Supplementary Information file. There are no restrictions on data availability.

Research involving human participants, their data, or biological material

Policy information about studies with human participants or human data. See also policy information about sex, gender (identity/presentation), and sexual orientation and race, ethnicity and racism.

Reporting on sex and gender	As the focus of the study is on the genetics of soil-transmitted helminths no patient-specific data or identifiers were included in any analyses performed.
Reporting on race, ethnicity, or other socially relevant groupings	As the focus of the study is on the genetics of soil-transmitted helminths no patient-specific data or identifiers were included in any analyses performed.
Population characteristics	As the focus of the study is on the genetics of soil-transmitted helminths no patient-specific data or identifiers were included in any analyses performed.
Recruitment	Parasites were opportunistically sampled from patients from different studies around the world. There was no specific recruitment criteria of the patients themselves to target specific parasites.

Ethics oversight

In all cases where parasites were collected from human patients, informed written consent was obtained from all participants or their guardians (in cases where the participant was a child) after being informed about the study in English and/or the local language.

Details on ethical approvals for the n=150 samples sequenced as part of this study is provided below:
Argentina (n = 1).
i. n = 1; whole genome sequencing data of DNA extracted from concentrated pools of eggs of all STH species; the original study protocol was approved by the bioethics committee of Colegio de Médicos de la Provincia de Salta and the IRBs of BCM (protocol number H-34926); citation: this study. Key collaborators: Nicolas R Caro, Ruben O Cimino, Alejandro J Krolewiecki, Rojelio Mejia.

Bangladesh (n = 10).
i. n = 10; whole genome sequencing data of DNA extracted from faecal samples; the original study protocol was approved by the Ethical Review Committee at icddr,b (PR-14105), the Committee for the Protection of Human Subjects at the University of California, Berkeley (2014-08-6658), and the institutional review board at Stanford University (27864); citation: this study. Key collaborators: John M Colford Jr, Jade Benjamin-Chung, Steven A Williams.

Benin (n = 25).
i. n = 25; whole genome sequencing data of DNA extracted from faecal samples, from the DeWorm3 project. One sample di not yield any sequencing reads. The original study protocol was approved by the Institut de Recherche Clinique au Bénin (IRCB) through the National Ethics Committee for Health Research (002-2017/CNERS-MS) from the Ministry of Health, the Human Subjects Division at the University of Washington (STUDY00000180) and the Data Safety and Monitoring Committee (DSMC); citation: this study. Key collaborators: Moudachirou Ibikounlé, Adrian JF Luty, Judd L Walson.

f. Democratic Republic of Congo (n = 1).
i .n = 1, whole genome sequencing data of DNA extracted from a faecal extract, part of the same protocol approval above. The original study protocol was approved by the Democratic Republic of Congo / University Hospital, Ghent University, Belgium (M104; Catholic University of Bukavu, Democratic Republic of the Congo [Ref: UCB/CIE/NC/016/2016], the Ministry

of Public Health, Democratic Republic of the Congo [Ref: 062/CD/DPS/SK/2017]); citation: this study.
Ethiopia (n =3)
ii. n = 2, whole genome sequencing data of DNA extracted from two A. lumbricoides worms. The original study protocol was approved by Ethical Review Committee, Faculty of Medicine and Health Sciences / University Hospital, Ghent University, Belgium [Ref: B670201627755 and PA2014/003], Jimma University, Ethiopia (Ref: RPGC/547/2016); citation: this study. Key collaborators: Piet Cools, Bruno Levecke, Zeleke Mekonnen.
iii. n = 1; whole genome sequencing data of DNA extracted from a faecal sample. The original study protocol was approved by the Ethical Review Committee, Faculty of Medicine and Health Sciences / University Hospital, Ghent University, Belgium (Ref: B670201627755 and PA2014/003), and by the Jimma University, Ethiopia (Ref: RPGC/547/2016); citation: this study. Key collaborators: Piet Cools, Bruno Levecke, Zeleke Mekonnen.

Fiji (n = 2).
i. n = 2, whole genome sequencing data of two aliquots of DNA extracted from a single A. lumbricoides worm; the worm was provided by the Natural History Museum, London, UK under registration number: 2012.11.19.1, Ascaris lumbricoides Linnaeus, 1758 -- Ascaridinae; Ascarididae; Ascaridoidea; Spirurina; Rhabditida; Chromadorea, 1, spirit material.

India (n = 25).
i. n = 25, whole genome sequencing data of DNA extracted from faecal samples, part of the DeWorm3 project. The original study protocol was approved by the Christian Medical College Institutional Review Board in Vellore, India (10392). The study was also approved by The Human Subjects Division at the University of Washington (STUDY00000180) and the genome skimming project was revised by the Data Safety and Monitoring Committee (DSMC); citation: this study. Key collaborators: Sitara SR Ajjampur, Malathi Manuel, Judd L Walson.

Kenya (n=7)
i. n = 7; whole genome sequencing data of DNA extracted from faecal samples; the original study protocol was approved by the Scientific and Ethics Review Committees (ERC) of the Kenya Medical Research Institute (KEMRI, SSC #1820); citation: (Onkanga et al., 2016; Secor et al., 2020). Key collaborators: Maurice Odiere, Pauline Mwinzi.

Malawi (n = 25).
i. n = 25; whole genome sequencing data of DNA extracted from faecal samples, part of the DeWorm3 project. The original study protocol was approved by The London School of Hygiene and Tropical Medicine (12013), The College of Medicine Research Ethics Committee (P.04/17/2161) in Malawi. The study was also approved by The Human Subjects Division at the University of Washington (STUDY00000180) and the genome skimming project was revised by the Data Safety and Monitoring Committee (DSMC); citation: this study. Key collaborators: Robin Bailey, David Chaima, Khumbo Kalua, Judd L Walson, Stefan Witek-McManus.

Myanmar (n = 32)
i. n = 32; whole genome sequencing data of DNA extracted from faecal samples; the original study protocol was approved by Imperial College London, UK (Ethical Review Ref: 17IC4249 and 17IC4249 NoA1); citation: this study. Key collaborators: Roy M Anderson, Julia Dunn.

Nigeria (n = 11).
i. n = 11; whole genome sequencing data of DNA extracted from faecal samples; the original study protocol was approved by the Health Research Ethics Committee of the Kebbi State Ministry of Health, Nigeria (reference number:105:23/2021); citation: this study. Key collaborator: Olumide Ajibola.

South Africa (n = 7)
i. n = 7; whole genome sequencing data of DNA extracted from faecal samples; the original study protocol was approved by the Biomedical Research Ethics Administration, University of KwaZulu-Natal KZN (Ref BF029/07); citation: this study. Key collaborator: Eyrun F Kjetland.

Sri Lanka (n = 1).
i. n = 1; whole genome sequencing data of DNA extracted from faecal samples. The original study protocol was approved by the Ethical Review Committee, Faculty of Medicine, University of Peradeniya, Sri Lanka [Ref: 2015/EC/58]; citation: this study. Key collaborators: Cinzia Cantacessi, Timothy P Jenkins.

Note that full information on the approval of the study protocol must also be provided in the manuscript.

Field-specific reporting

Please select the one below that is the best fit for your research. If you are not sure, read the appropriate sections before making your selection.

- [x] Life sciences
- [ ] Behavioural & social sciences
- [ ] Ecological, evolutionary & environmental sciences

For a reference copy of the document with all sections, see nature.com/documents/nr-reporting-summary-flat.pdf

Life sciences study design

All studies must disclose on these points even when the disclosure is negative.

Sample size	No sample size calculations were used in the study design. Samples were chosen to maximise the number of populations in geographically distinct regions of the world. A total of 1000 parasite and faecal samples were analysed from 27 countries, a mix of samples sequenced de novo on this study and data obtained from the European Nucleotide Arc
Data exclusions	Data were filtered at different stages for different reasons, for example, to remove low quality SNPs, to ensure all SNPs were present in some or all samples depending on the analysis, and that SNPs had sufficient coverage to be accurately called. The steps and parameters used are described in detail in both the methods and the code made available.
Replication	As this was an exploration of genetic diversity, there were no attempts for data replication.
Randomization	There was no grouping of samples, other than by the country, population from where they were collected and/or life stage of the worms.
Blinding	As this study focused on describing the genetic diversity of parasites within and between specific locations, blinding of samples was not relevant

Reporting for specific materials, systems and methods

We require information from authors about some types of materials, experimental systems and methods used in many studies. Here, indicate whether each material, system or method listed is relevant to your study. If you are not sure if a list item applies to your research, read the appropriate section before selecting a response.

Materials & experimental systems

n/a	Involved in the study	
x		Antibodies
x		Eukaryotic cell lines
x		Palaeontology and archaeology
	x	Animals and other organisms
		Clinical data
x		Dual use research of concern
x		Plants

Methods

n/a	Involved in the study	
x		ChIP-seq
x		Flow cytometry
x		MRI-based neuroimaging

Animals and other research organisms

Policy information about studies involving animals; ARRIVE guidelines recommended for reporting animal research, and Sex and Gender in Research

Laboratory animals	The study did not involve laboratory animals
Wild animals	The study did not involve laboratory animals
Reporting on sex	The study did not choose samples based on sex but merely on parasitic load.
Field-collected samples	The study did not concern field-collected samples. All samples had been collected prior to this work with all relevant paperwork and ethical clearances in place.
Ethics oversight	No ethical guidance or approval was necessary to work on the parasites themselves. Samples were either compliant or exempt from the Nagoya Protocol. All relevant amendments to existing protocols have been provided in the Supplementary Information document and under the Methods section.

Note that full information on the approval of the study protocol must also be provided in the manuscript.

Clinical data

Policy information about clinical studies

All manuscripts should comply with the ICMJE guidelines for publication of clinical research and a completed CONSORT checklist must be included with all submissions.

Clinical trial registration	*Provide the trial registration number from ClinicalTrials.gov or an equivalent agency.*
Study protocol	*Note where the full trial protocol can be accessed OR if not available, explain why.*
Data collection	*Describe the settings and locales of data collection, noting the time periods of recruitment and data collection.*
Outcomes	*Describe how you pre-defined primary and secondary outcome measures and how you assessed these measures.*

Plants

Seed stocks	*Report on the source of all seed stocks or other plant material used. If applicable, state the seed stock centre and catalogue number. If plant specimens were collected from the field, describe the collection location, date and sampling procedures.*
Novel plant genotypes	*Describe the methods by which all novel plant genotypes were produced. This includes those generated by transgenic approaches, gene editing, chemical/radiation-based mutagenesis and hybridization. For transgenic lines, describe the transformation method, the number of independent lines analyzed and the generation upon which experiments were performed. For gene-edited lines, describe the editor used, the endogenous sequence targeted for editing, the targeting guide RNA sequence (if applicable) and how the editor was applied.*
Authentication	*Describe any authentication procedures for each seed stock used or novel genotype generated. Describe any experiments used to assess the effect of a mutation and, where applicable, how potential secondary effects (e.g. second site T-DNA insertions, mosiacism, off-target gene editing) were examined.*
